# Altered structural connectome in non-lesional newly diagnosed focal epilepsy: Relation to pharmacoresistance

**DOI:** 10.1016/j.nicl.2021.102564

**Published:** 2021-01-19

**Authors:** Barbara A.K. Kreilkamp, Andrea McKavanagh, Batil Alonazi, Lorna Bryant, Kumar Das, Udo C. Wieshmann, Anthony G. Marson, Peter N. Taylor, Simon S. Keller

**Affiliations:** a Department of Pharmacology & Therapeutics, Institute of Systems, Molecular and Integrative Biology, University of Liverpool, Liverpool, UK; b Department of Neuroradiology, The Walton Centre NHS Foundation Trust, Liverpool, UK; c Department of Clinical Neurophysiology, University Medicine Göttingen, Göttingen, Germany; d Department of Radiology and Medical Imaging, Prince Sattam Bin Abdulaziz University, Al Kharj, Saudi Arabia; e CNNP Lab, Interdisciplinary Computing and Complex BioSystems Group, School of Computing, Newcastle University, UK; f UCL Queen Square Institute of Neurology, Queen Square, London, UK

**Keywords:** Diffusion MRI, Network based statistics, Anti-epileptic drug outcome

## Abstract

Despite an expanding literature on brain alterations in patients with longstanding epilepsy, few neuroimaging studies investigate patients with newly diagnosed focal epilepsy (NDfE). Understanding brain network impairments at diagnosis is necessary to elucidate whether or not brain abnormalities are principally due to the chronicity of the disorder and to develop prognostic markers of treatment outcome. Most adults with NDfE do not have MRI-identifiable lesions and the reasons for seizure onset and refractoriness are unknown. We applied structural connectomics to T1-weighted and multi-shell diffusion MRI data with generalized q-sampling image reconstruction using Network Based Statistics (NBS). We scanned 27 patients within an average of 3.7 (SD = 2.9) months of diagnosis and anti-epileptic drug treatment outcomes were collected 24 months after diagnosis. Seven patients were excluded due to lesional NDfE and outcome data was available in 17 patients. Compared to 29 healthy controls, patients with non-lesional NDfE had connectomes with significantly decreased quantitative anisotropy in edges connecting right temporal, frontal and thalamic nodes and increased diffusivity in edges between bilateral temporal, frontal, occipital and parietal nodes. Compared to controls, patients with persistent seizures showed the largest effect size (|d|>=1) for decreased anisotropy in right parietal edges and increased diffusivity in edges between left thalamus and left parietal nodes. Compared to controls, patients who were rendered seizure-free showed the largest effect size for decreased anisotropy in the edge connecting the left thalamus and right temporal nodes and increased diffusivity in edges connecting right frontal nodes. As demonstrated by large effect sizes, connectomes with decreased anisotropy (edge between right frontal and left insular nodes) and increased diffusivity (edge between right thalamus and left parietal nodes) were found in patients with persistent seizures compared to patients who became seizure-free. Patients who had persistent seizures showed larger effect sizes in all network metrics than patients who became seizure-free when compared to each other and compared to controls. Furthermore, patients with focal-to-bilateral tonic-clonic seizures (FBTCS, N = 11) had decreased quantitative anisotropy in a bilateral network involving edges between temporal, parietal and frontal nodes with greater effect sizes than those of patients without FBTCS (N = 9). NBS findings between patients and controls indicated that structural network changes are not necessarily a consequence of longstanding refractory epilepsy and instead are present at the time of diagnosis. Computed effect sizes suggest that there may be structural network MRI-markers of future pharmacoresistance and seizure severity in patients with a new diagnosis of focal epilepsy.

## Highlights

• Patients showed showed widespread connectome alterations relative to controls.
• Relative to controls, patients /w seizure-freedom (SF) had increased diffusivity.
• Patients /w persistent seizures (PS) had increased diffusivity relative to controls.
• Subgroup-specific connectomes were found for both patient groups (SF vs PS).
• Patients with generalized seizures and those without had altered connectomes.

## 1 Introduction

Compared to well-studied longstanding epilepsy, sophisticated neuroimaging studies in patients with newly diagnosed focal epilepsy (NDfE) are scarce. Understanding brain network impairments at diagnosis is necessary to elucidate whether or not brain abnormalities are a consequence of longstanding refractory epilepsy. Furthermore, the study of focal epilepsy at the earliest time-point could allow the development of prognostic markers of treatment outcome. This is critical for patients, as early appropriate treatment is more likely to increase the likelihood of seizure freedom (Kwan and Brodie, 2000). Understanding how the brain is altered in the earliest stages of human epilepsy will likely yield important MRI-markers associated with biological processes underlying the disorder, seizure outcome and comorbidities. An early marker of pharmacoresistance could enable selected patients to be re-routed to alternative or adjunctive treatments at an earlier time-point, which would save time, costs and chronic exposure to persistent side-effects of anti-epileptic drugs (AEDs).

The vast majority of adults with NDfE have no MRI-identifiable lesion (Liu et al., 2002, Van Paesschen et al., 1997). There are limited insights from diagnostic MRI and EEG on how the brain is perturbed at the time of epilepsy diagnosis (Pohlmann-Eden et al., 2013). Many studies do not reveal cohort-based volumetric alterations in patients with NDfE based using conventional quantitative analysis (Liu et al., 2001, Liu et al., 2002, Hagemann et al., 2002, Salmenpera et al., 2005, Alonazi et al., 2019). A recent study in patients with NDfE reported reduced volume within the corpus callosum in patients who became seizure-free when compared to patients who did not respond to AEDs (Kim et al., 2017), although this did not survive correction for multiple comparisons. One reason why volumetric MRI studies are unrevealing in NDfE may be because the pathological brain changes that result from the development of epileptogenic brain networks are not amenable to detection through analysis of gross brain morphometry. It is therefore important to investigate patients with more advanced imaging techniques that provide insights into the perturbed brain networks at the time of diagnosis (Pohlmann-Eden et al., 2013, de Bézenac et al., 2019).

Epilepsy is a system disorder with abnormal short- and long-range inter-ictal network connectivity, as has been shown with electrophysiological and functional MRI recordings (Blumenfeld, 2014, Wykes et al., 2019). As structures within an epileptogenic network are involved in generation and expression of seizures and may contribute to the maintenance and refractoriness of the disorder, neuroimaging approaches should endeavour to model these networks. Structural brain networks are known to be affected in patients with longstanding refractory focal epilepsy and may provide novel prognostic markers of postsurgical outcome (Taylor et al., 2018, Bonilha et al., 2015, Bonilha et al., 2013). For these reasons, analysis of brain networks is gathering pace in patients with chronic epilepsy (Bernhardt et al., 2015). However, it is not known if structural network changes are driven by the chronicity of epilepsy, including the long-term use of AEDs, and the potentially deleterious effects of recurrent seizures. These issues could be resolved by using novel applications of network analysis (connectomics) in patients with NDfE.

Focal onset epilepsy may become pharmacoresistant (Kwan and Brodie, 2000), may be associated with memory dysfunction (van Rijckevorsel, 2006) and can impact on patient quality of life. Approximately 60% of patients with NDfE will have seizure remission with AED treatment, while the remainder may continue to experience seizures (Brodie, 2013, Mohanraj and Brodie, 2013). Fewer seizures before the commencement of AED treatment, response to the first prescribed AED, genetic-generalized epilepsy and the absence of an MRI-identifiable lesion have been related to improved AED treatment outcomes (Kwan and Brodie, 2000). There are, however, no markers of pharmacoresistance that can stratify individual patients at the time of diagnosis. Like studies addressing surgical outcome in refractory focal epilepsy (Taylor et al., 2018, Bonilha et al., 2015, Bonilha et al., 2013), network-based approaches may provide unique *in-vivo* imaging prognostic markers of AED treatment outcome.

In the present study we investigated structural network alterations in patients with non-lesional NDfE relative to healthy controls. DSI-studio (http://dsi-studio.labsolver.org) allows deterministic tractography of multi-shell diffusion MRI using a generalized q-sampling imaging technique which is more advanced than the more commonly used diffusion tensor imaging technique (Yeh et al., 2010). Q-sampling affords the advantage of identifying kissing and crossing fibers by virtue of estimating the spin distribution function directly from diffusion MRI giving a more accurate representation of the underlying biology (Fan et al., 2016). Network Based Statistics (NBS, Zalesky et al., 2010) implements statistical methods for hypothesis-testing on human connectomes and has identified network alterations in other disorders such as depression (Tymofiyeva et al., 2017), schizophrenia (Zalesky et al., 2011, Cocchi et al., 2014) and other neurodegenerative disorders (Gou et al., 2018, Wang et al., 2019a, Wang et al., 2019b). NBS offers multiple-comparison corrected whole-brain connectomics with sensitive assessment of large-scale structural network alterations. In order to determine whether structural networks have potential as an MRI-marker of pharmacoresistance, we also collected two-year AED treatment outcomes after diagnosis and explored whether network alterations were related to treatment outcome.

## 2 Methods

### 2.1 Participants

We studied 27 adults with NDfE (mean age = 33, SD = 11, 12 female) and 29 healthy controls (mean age = 32, SD = 11, 16 female). Our study received prior approval by the local research ethical committee (reference 14/NW/0332) and informed consent was obtained from each participant. Patients with NDfE were recruited from the Walton Centre NHS Foundation Trust, Liverpool, UK. Patients were scanned an average 3.7 months after diagnosis (SD 2.9, range 1–11 months, Alonazi et al., 2019). Focal epilepsy was diagnosed by expert epileptologists based on ILAE classifications and seizure semiology. Seizure history was recorded (focal seizures without and with impaired awareness, focal-to-bilateral tonic-clonic seizures, focal seizures without and with impaired awareness with focal-to-bilateral tonic-clonic seizures). Patients with primary generalized seizures, provoked seizures (e.g. drug induced), acute symptomatic seizures (e.g. brain injury) or known progressive neurological disease (e.g. brain tumor) or other neurological/psychiatric conditions were excluded. None of the controls had a history of neurological/psychiatric conditions. Controls and patients were comparable in age and sex. Seven patients were excluded from analysis due to a neuroradiologically confirmed lesion (Alonazi et al., 2019) that would have biased connectome computation and lead to underpowered analyses. 24-month outcomes were collected; nine patients had persistent seizures and eight patients were seizure free. Outcome data was unavailable in three patients as these did not have recent clinical appointments. All demographic and clinical information is presented in Table 1.

**Table 1:** Demographic and clinical information for all study participants.

	**Controls**	**Patients**	
**Demographic Information**			
N	29	27	
Mean age ± SD in years	32 ± 12	33 ± 11	
Sex (female / male)	16 / 13	12 / 15	

**Clinical Information**		**Lesional**	**Non-lesional**

N	–	7	20
EEG (normal / abnormal)	–	7 / 0	17 / 3
FSA (yes / no)	–	0 / 7	2 / 18
FSIA (yes / no)	–	0 / 7	3 / 17
FBTCS (yes / no)	–	5 / 2	11 / 9
FBTCS: At 24 months (PS / SF / NO)	–	1 / 4 / 0	3 / 6 / 2
At 24 months (PS / SF / NO)	–	3 / 4 / 0	9 / 8 / 3

*Note.* FSA = focal seizure with awareness; FSIA = focal seizure with impaired awareness; FBTCS = focal-to-bilateral tonic-clonic seizure; EEG = electro-encephalography; PS = persistent seizures; SF = seizure-free; NO = no outcomes at 24 months.

### 2.2 MRI acquisition

Participants were scanned at the Liverpool Magnetic Resonance Imaging Centre (LiMRIC) on 3 T MR (Siemens Trio). We acquired an isotropic 1 mm T1-weighted (T1w, Magnetization Prepared Rapid Gradient Echo sequence with 176 axial slices, TE = 5.57 ms, TR = 2040 ms, TI = 1100 ms, flip angle = 8°), isotropic 1 mm T2-weighted (T2w, Turbo Spin Echo sequence with 160 axial slices, TE = 355 ms, TR = 3000 ms) and isotropic 3.1 mm multi-shell diffusion MRI (dMRI) with b-values of 1000 and 2000 s/mm^2^, 60 gradients each and a b0 image (72 axial slices, TE = 104 ms, TR = 5.7 ms, flip angle = 90°, acceleration factor = 2).

### 2.3 Preprocessing

**Fig. 1:**
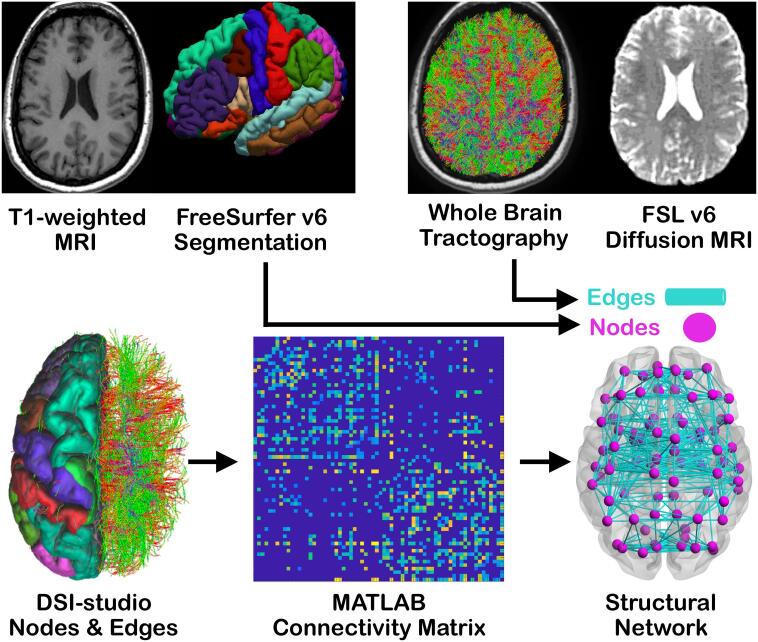
**Processing steps shown on an example participant.** Data was processed with FreeSurfer (T1w), FSL (dMRI), DSI-studio (combined nodes and edges), MATLAB (connectivity matrix) and NBS (structural network). As seen from left to right and top to bottom. T1w = T1-weighted MRI; dMRI = diffusion MRI; NBS = network-based statistics.

All preprocessing steps are shown in Fig. 1 on the basis of one participants' data. Briefly, connectomes were computed from T1w and multi-shell dMRI data. For generation of T1w gray matter segments, cortical and subcortical parcellations were computed using Freesurfer Version 6 with the Desikan-Killiany atlas (Fischl et al., 2002). As the size of parcellated regions within an atlas may influence connectomics, we have applied the same analysis on an additional atlas (Destrieux et al., 2010) and results are summarized and presented in supplementary materials. The gray and white matter boundary segmentations were manually corrected using the recommended FreeSurfer PialEdits/ControlPoints procedures before 82 regions-of-interest were extracted and used as network nodes for connectomics (Taylor et al., 2018). The dMRI data was preprocessed using FMRIB Software Library (FSL) version 6 (http://fsl.fmrib.ox.ac.uk/fsl/fslwiki/FSL, Smith et al., 2004) according to the ENIGMA dMRI-preprocessing steps to mitigate effects of image artifacts (http://enigma.ini.usc.edu/protocols/dti-protocols/), such as echo-planar image distortions (Andersson et al., 2003), as published in our previous work (Kreilkamp et al., 2019). Briefly, the b0 images were brain-extracted and motion-corrected, while distortion-correction was achieved by the use of a brain-extracted T2w image. The resulting mean b0 image served as a reference volume for motion- and distortion-correction on the diffusion-weighted images and the gradient table was rotated according to the motion-parameters (Leemans and Jones, 2009). The root-mean-square values (unit in millimeters) representing the mean translational and rotational movement across all intra-cerebral voxels from all diffusion-weighted images relative to the first b0 image were extracted from the FSL eddy output files 'eddy_movement_rms'. We computed the mean of these values across all diffusion-weighted images for every participant to perform group-wise comparisons between all patients, patients with persistent seizures, patients who became seizure-free, patients with FBTCS, patients without FBTCS and controls.

For generation of white matter connections (edges), DSI-studio (Build 27-02-2019, Yeh et al., 2019) was used to perform deterministic tractography using a higher-order q-sampling imaging (Yeh et al., 2010) rather than a more simplistic diffusion-tensor reconstruction approach. DSI-studio has been demonstrated to provide the most reliable reconstruction of valid connections (Maier-Hein et al., 2017). Data were reconstructed using three shells (B = 0; B = 1000 and B = 2000 s/mm^2^). The diffusion data were upsampled to isotropic 1.55 mm to allow tractography (Ahmadi et al., 2009, Yeatman et al., 2012) and fibers were reconstructed using generalized q-sampling imaging with a diffusion sampling length ratio of 1.25. The orientation distribution function calculation was weighted by the square of the diffusion displacement. The step size was 1 mm and quantitative anisotropy (QA) and angular threshold were set to 0.1 and 60 degrees respectively. A total of 5,000,000 streamlines longer than 10 mm and shorter than 300 mm were calculated. Matrix entries represented edges from tractography and were set as present if more than one streamline terminated in two nodes derived from segmentations. The average edge diffusion metrics were directly calculated from all streamlines connecting two nodes. Connectivity matrix thresholding was performed so that edges were present in at least 75% of all controls/patients (most common in sample) and edges were common to every group (Besson et al., 2014a).

### 2.4 Statistics

All statistical analyses were performed using MATLAB 2018b. Age was normally distributed in patients and controls (Lilliefors test p > 0.05), we therefore used an unpaired *t*-test when testing for differences in age of controls and patients. A chi-square test of independence was used for analysis of sex distribution differences in controls and patients. We also conducted a chi-square test of independence with Yates-correction for small sample sizes to calculate whether patients with FBTCS (N = 3 in PS) and those without FBTCS (N = 6 in SF) were equally distributed across groups of patients with persistent seizures and those who became seizure-free. The average root-mean-square motion values computed from diffusion-weighted images were tested for normality using a Lilliefors test (data non-normally distributed for all groups at p < 0.05) and analyzed with an unpaired Mann-Whitney *U* test to compare controls and patients and a Kruskal-Wallis ANOVA for patients with persistent seizures, patients who became seizure-free and controls. For patient and control group network analysis we performed an unpaired *t*-test within NBS v1.2 running 10,000 permutations for multiple comparison correction. NBS accounts for controlling Type I errors by reducing the number of comparisons based on initial T-score thresholds followed by permutation testing for family-wise error correction (Zalesky et al., 2012). It is therefore necessary to validate significant findings across multiple T-score thresholds. T-score thresholds were set to 1.5–4.0 in increments of 0.1 to investigate significant effects in diffusion networks. We used this range of T-score thresholds in NBS to validate the robustness of significant findings when comparing patients and controls. We have not performed an analysis of streamline count since this measure may be influenced by gray matter parcel size. Furthermore, this measure may depend on tractography algorithm, curvature, length, width and myelination and may not be considered a good indicator for fiber count (Jones et al., 2013). We therefore chose to analyze diffusion metrics that will be less affected by parcel size, are commonly analyzed in the literature and may provide a better representation of structural alterations than streamline count. The diffusion metrics analyzed included fractional/quantitative anisotropy (FA/QA) for measurement of diffusion directionality based on anisotropic diffusion within voxels; mean diffusivity (MD) quantifying the average magnitude of diffusion in all directions; radial/axial diffusivity (RD/AD) which provide indication on the magnitude of diffusion radially and parallel to the principle direction of diffusion in every voxel. Age and sex were used as covariates in all analyses. Spearman correlations between clinical characteristics (age of onset, seizure type and seizure frequency between diagnosis and MRI) and the average of significantly altered diffusion metrics within the patient versus control networks were investigated. Results were considered significant at p < 0.05. For visualizing NBS networks we have selected the exemplary threshold of |T|>=2.7, corresponding to two-tailed p = 0.0096 (patients N = 20; controls N = 29; 48 degrees of freedom). This threshold was chosen as an example due to its high significance level in our sample while all other T-scores and edges are shown in the figures and in supplementary materials.

In order to determine the sample size needed to detect significant effects when comparing patients who became seizure-free, patients with persistent seizures and controls, we conducted an a priori power analysis using G*Power3 (Faul et al., 2007). Sufficiently powered sample sizes were computed for a one-way fixed-effects ANOVA between three groups with a large effect size (|d|>=1) and an alpha value of 0.05. Results showed that a total sample of 42 participants with three equally sized groups of N = 14 would be required to achieve a power of at least 0.80. As the power analysis indicated that we were underpowered to perform an ANOVA in NBS for subgroup analysis (patients with persistent seizures N = 9; patients who were seizure-free N = 8; patients with FBTCS N = 11; patients without FBTCS N = 9), we computed Cohen's D effect sizes corrected for small samples based on the estimated marginal means corrected for age and sex. The magnitude of Cohen's D effect sizes were interpreted following previously published criteria: d (0.01–0.19) = very small, d (0.2–0.49) = small, d (0.5–0.79) = medium, d (0.8–1.19) = large, d (1.2–1.99) = very large, and d (>=2.0) = huge (Sawilowsky, 2008). We report effect sizes with magnitudes of at least |d|>=1.

## 3 Results

Analysis of demographic information did not reveal any significant difference in age between patients and controls (t_(48)_ = −0.2799, *p* = 0.78) or sex (χ^2^_(1)_ = 0.64, *p* = 0.42). The number of patients with FBTCS and those without FBTCS were equally distributed within groups of patients with persistent seizures and those who became seizure-free: χ^2^_Yates (1)_ = 1.5, *p*_Yates_ = 0.22.

The descriptive statistics for root-mean-square motion values computed from the diffusion-weighted images were as follows: controls (Mean ± SD = 1.23 ± 0.41), patients (Mean ± SD = 1.42 ± 1.32), patients with persistent seizures (Mean ± SD = 1.84 ± 1.93), patients who were seizure free (Mean ± SD = 1.03 ± 0.20), patients with FBTCS (Mean ± SD = 1.62 ± 1.77) and patients without FBTCS (Mean ± SD = 1.18 ± 0.27). Unpaired Mann-Whitney *U* test between controls and patients (Z = −0.50, *p* = 0.62), patients with FBTCS and those without FBTCS (Z = −0.46, *p* = 0.65) and Kruskal-Wallis ANOVA between the two patient subgroups (SF / PS) and controls (χ^2^(2) = 2.57, *p* = 0.28) did not reveal any significant differences.

**Fig. 2:**
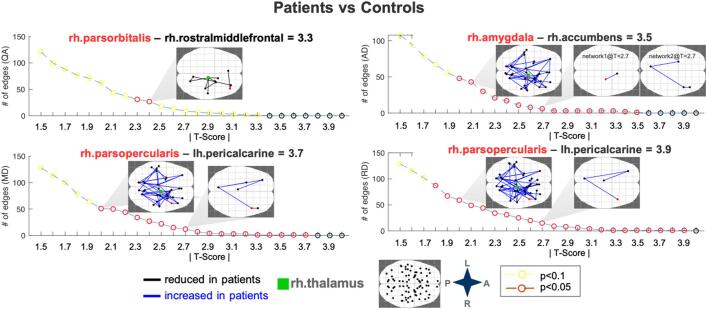
**Significant NBS networks in patients vs controls across different T-score thresholds.** Networks were visualized with NBSview. Inset shows complete node set used for analysis. NBS identified several edges for different T-scores and significant networks are displayed for selected exemplary T-scores for visualization purposes. Edges with the highest T-scores are denoted by highlighting the affected node in red along with the connected node and T-score in the subplot titles. L = left; R = right; A = anterior; P = posterior; rh = right hemisphere; lh = left hemisphere; QA = quantitative anisotropy; MD/AD/RD = mean/axial/radial diffusivity. (For interpretation of the references to color in this figure legend, the reader is referred to the web version of this article.)

**Fig. 3:**
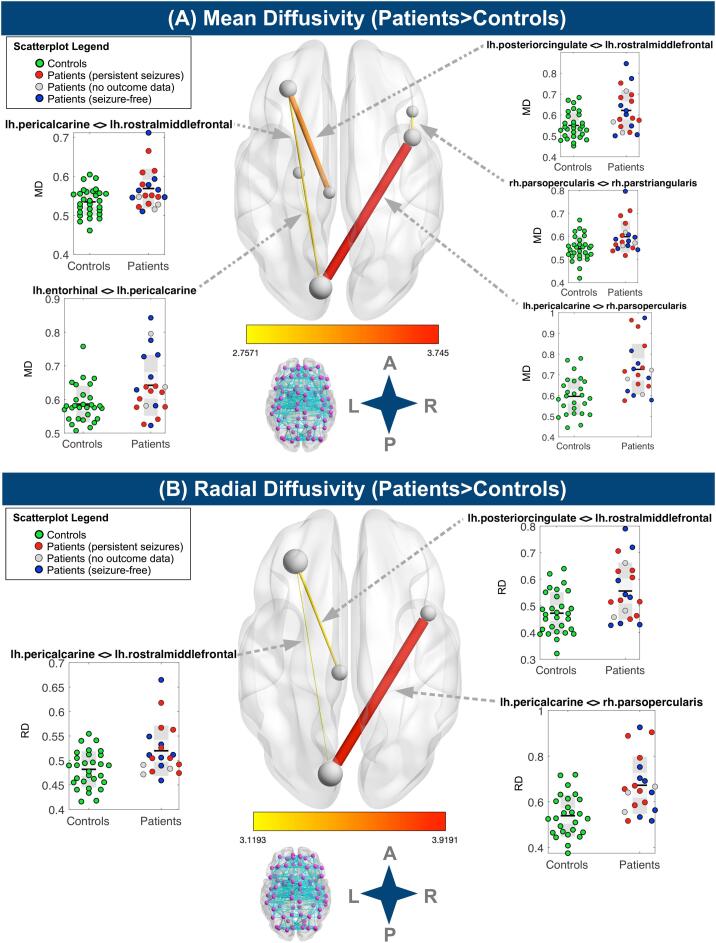
**Significant mean (A) and radial diffusivity (B) connectograms (|T|>=2.7) with scatterplots.** All diffusivity metrics are in units of 10^−3^ mm^2^/s. Networks were visualized with BrainNet Viewer (Xia et al., 2013). Node size represents number of edges. Edge thickness and color represent magnitude of T-statistic. Inset shows complete node set (N = 82) with edges used for analysis. L = left; R = right; A = anterior; P = posterior; MD/RD = mean/radial diffusivity.

Patient brain networks showed decreased anisotropy and increased diffusivity (QA range = 2.3–2.4; AD range = 2.0–3.5; MD range = 2.0–3.7; RD range = 1.8–3.9) across multiple NBS thresholds relative to controls (Fig. 2), but no alterations in FA. Our supplementary analysis using the Destrieux atlas also showed decreased QA and increased MD and RD across multiple NBS thresholds in patients relative to controls (Fig. 1, Suppl), but no alterations in FA or AD. Within the Desikan-Killiany atlas, the edges with the largest absolute T scores (|T|) for every metric are highlighted in red in Fig. 2 and involved the right pars orbitalis, right rostral middle frontal, right pars opercularis, right amygdala, right nucleus accumbens and left pericalcarine nodes. The right thalamus showed altered edges in all networks (|T|>=2.4 for decreased QA and |T|>=2.0 for increased MD/RD/AD; Fig. 2). There was also significantly increased AD within a separate network between right amygdala and right nucleus accumbens. Fig. 3 shows MD and RD value distributions in controls and patients (where patients were color-coded for outcome) for networks with significant alterations. There were no significant correlations between clinical variables (age of onset, history of seizure type and seizure frequency) and network diffusion metrics.

**Fig. 4:**
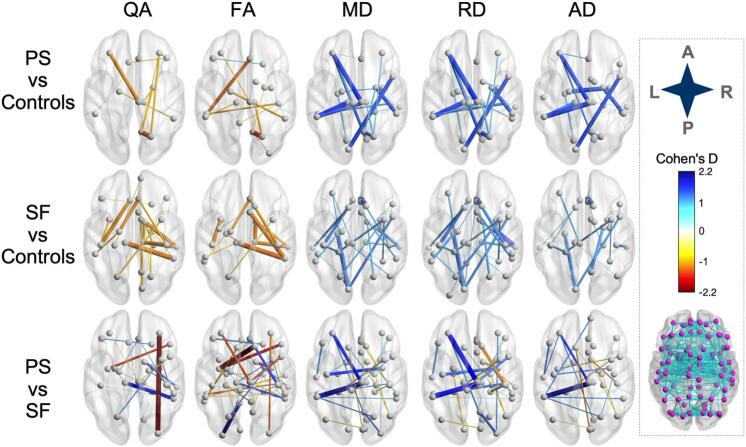
**NBS networks with small-sample-size corrected Cohen's Dmagnitudes** **> 1.0 in outcome subgroup comparisons.** Networks were visualized with BrainNet Viewer using the red-white-blue colormap (He, 2020). Edge thickness and color represent magnitude of Cohen's D. Inset shows complete node set with edges used for analysis. SF = seizure-free; PS = persistent seizures; L = left; R = right; A = anterior; P = posterior; QA/FA = quantitative/fractional anisotropy; MD/AD/RD = mean/axial/radial diffusivity. (For interpretation of the references to color in this figure legend, the reader is referred to the web version of this article.)

Patients who went on to experience persistent seizures showed increased diffusivity and decreased QA and FA for multiple edges as demonstrated by the Cohen's d values (QA min = −1.4 ; FA min = −1.6; MD max = 1.6; RD max = 1.6; AD max = 1.6) relative to controls. The edges with the largest absolute Cohen's d values (|d|) are presented in Fig. 4, Table 1 (Suppl) and included bilateral parietal and left thalamic nodes. There were also two edges that demonstrated increased FA compared to controls (edge connecting the right lateral orbitofrontal node with the left pars orbitalis node and edge between the right lateral orbitofrontal node and right banks of the superior temporal sulcus node).

Patients who were seizure free had networks with increased diffusivity and decreased QA and FA for multiple edges (Cohen's d: QA min = −1.4; FA min = −1.42; MD max = 1.5; RD max = 1.5; AD max = 1.5) relative to controls (Fig. 4, Table 1, Suppl). There was also a single edge that demonstrated a large |d| for FA compared to controls (edge connecting the right superior frontal node with the left insula node). All details on edges with large |d| are presented in Table 1 (Suppl) and included left thalamic and right temporal and frontal nodes.

Patients who went on to experience persistent seizures had networks with increased diffusivity and decreased QA and FA for multiple edges (Cohen's d: QA min = −2.3; FA min = −2.5; MD max = 2.1; RD max = 1.8; AD max = 2.5) relative to patients who became seizure-free (Fig. 4, Table 1, Suppl). Conversely, patients who became seizure-free had networks with increased diffusivity and decreased QA and FA for a different set of edges (Cohen's d values: QA max = 2; FA max = 2.3; MD min = −1.2; RD min = −1.5; AD min = −1.4). All details on edges with large |d| are presented in Table 1 (Suppl) and included right frontal, left insular/parietal and right thalamic nodes. Patients who had persistent seizures showed larger effect sizes in all network metrics than patients who became seizure-free when compared to each other and compared to controls.

**Fig. 5:**
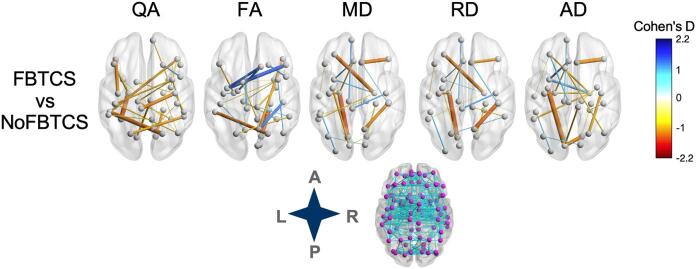
**NBS networks with small-sample-size corrected Cohen's Dmagnitudes> 1.0 in FBTCS subgroup comparisons.** Networks were visualized with BrainNet Viewer using the red-white-blue colormap. Edge thickness and color represent magnitude of Cohen's D. Inset shows complete node set with edges used for analysis. SF = seizure-free; PS = persistent seizures; L = left; R = right; A = anterior; P = posterior; QA/FA = quantitative/fractional anisotropy; MD/AD/RD = mean/axial/radial diffusivity. (For interpretation of the references to color in this figure legend, the reader is referred to the web version of this article.)

Patients with FBTCS had decreased quantitative anisotropy in a bilateral temporal, parietal and frontal connectome and increased diffusivity values when compared to patients without FBTCS in inter-hemispheric temporal and frontal edges (Fig. 5, Table 2, Suppl). Compared to patients without FBTCS, patients with FBTCS had 57 edges with |d|>1.0. The largest decrease in QA and FA was found in an edge connecting the left fusiform with left entorhinal node and between right parahippocampal and right lingual nodes, respectively. The edge connecting right precentral and left paracentral nodes showed the largest |d| value in MD and RD for patients with FBTCS. The edge with the largest increase in AD was between the right entorhinal and left temporal pole nodes.

Compared to patients with FBTCS, patients without FBTCS had 50 edges with |d|>1.0 (Fig. 5, Table 2, Suppl). The largest decrease in QA and FA was found in an edge connecting the right pars opercularis and right lateral orbito frontal node and between the right pastriangularis and left insula node, respectively. The left lingual and left entorhinal edge showed the largest MD and RD effect size increases while the left insula and left pericalcarine edge had the largest increase in AD.

## 4 Discussion

Our results indicate that patients with non-lesional focal epilepsy have bi-hemispheric structural network alterations at the time of diagnosis at a group level. Additionally, we have demonstrated large effect size differences in bilateral structural networks between patients who became seizure-free, patients with persistent seizures and controls. We further demonstrate increased bi-hemispheric structural network alterations in patients with FBTCS to patients without FBTCS. This may suggest that at least some of the structural network alterations reported in patients with refractory focal epilepsy may not necessarily emerge as a consequence of long-term epilepsy and some may relate to AED treatment outcome and the presence of FBTCS seizures. Bilateral network alterations seen in our sample are similar to what is reported in some studies of patients with refractory focal epilepsy. Patients with longstanding focal epilepsy are typically electrophysiologically well-characterized, allowing ipsi-/contralateral analysis, as opposed to those with a new onset of seizures for whom localization information frequently is not readily available. Our study suggests that connectome alterations are already present in NDfE.

Studying patients with NDfE provides a unique opportunity to determine brain alterations at the earliest time in human epilepsy (Pohlmann-Eden et al., 2013, de Bézenac et al., 2019). This approach is more favorable than extrapolating information from cross-sectional correlational analyses based on duration of epilepsy and MRI-derived measures of pathology as commonly done when studying refractory focal epilepsy (Keller et al., 2015, Kreilkamp et al., 2019). Some quantitative MRI studies in patients with NDfE have not found cohort-based hippocampal or cortical atrophy (Liu et al., 2002, Salmenpera et al., 2005, Alonazi et al., 2019). Advanced imaging techniques, which may include diffusion MRI connectivity and network approaches, are in short supply in NDfE (Pohlmann-Eden et al., 2013). Park et al. (2020) described a graph-theoretical analysis of conventional diffusion-tensor imaging and reported that patients with persistent seizures had significantly lower assortativity coefficient values when compared to patients who became seizure-free. According to the authors a lower assortativity coefficient makes the network more vulnerable to attack (Park et al., 2020). This is consistent with our results that indicate reduced anisotropy and increased diffusivity in edges of patients with persistent seizures with larger effect sizes relative to those found in patients that were rendered seizure-free. Using multi-shell diffusion MRI, we report significant network alterations in patients relative to controls. We have shown that compared to controls, all patients had altered structural connections between frontal (left rostral middle frontal), temporal (left entorhinal) and limbic (left posterior cingulate) nodes.

Patients had bilateral networks with decreased QA and increased AD/MD/RD but unchanged FA, which may suggest increased inter-axonal space due to altered myelination or white matter atrophy (Nagy et al., 2016). Compared to FA, QA makes use of both non-zero b-shells in our data, is less sensitive to the partial volume effects of crossing fibers and free water, suggesting that it is a robust index for reproducible tractography (Yeh et al., 2013). This distinct methodological nature can explain the discrepant results between FA and QA values. Our findings here suggest, that in patients with NDfE QA is a more sensitive marker than FA and can be useful when determining presence of FBCTS at this early stage of epilepsy. In contrast to this, when comparing patients with PS to patients who were rendered seizure-free, FA showed more edges and edges with larger effect sizes compared to QA. However, as noted earlier, the FA metric may include more partial volume effects due to crossing fibers and free water than QA.

The thalamus, which has important roles in seizure initiation, propagation and modulation of focal seizures (Blumenfeld et al., 2009, Guye et al., 2006, Filipescu et al., 2019, Wykes et al., 2019, Keller et al., 2015, Caciagli et al., 2020, He et al., 2020), may be a crucial structural hub as an imaging marker of pharamacoresistance or FBTCS. Studies in genetic generalized epilepsy have demonstrated altered thalamic connectivity (Sinha et al., 2019, Wang et al., 2019a, Wang et al., 2019b), which measured shortly after diagnosis, could be related to AED treatment outcomes (Wang et al., 2019a, Wang et al., 2019b). Longstanding focal epilepsy with poor post-surgical outcome has been linked to altered thalamocortical connections using probabilistic tractography (Keller et al., 2015) and connectomics where temporal and parietal networks have been shown to be altered in patients with persistent seizures (Bonilha et al., 2013). Furthermore, the insula has been implicated in temporal plus surgical failures (Barba et al., 2017). Compared to all other groups, patients who had persistent seizures showed larger effect sizes in all network metrics. Previous research in longstanding focal epilepsy has shown that more widespread brain connectivity alterations are associated with surgical refractoriness, which may be a marker of a wider epileptogenic network in those with persistent postoperative seizures (Keller et al., 2015, Bonilha et al., 2013). The present study is the first to report wider network alterations in pharmacoresistant patients at the time of diagnosis of focal epilepsy. Importantly, the largest effect size difference for patients who had persistent seizures relative to patients who were rendered seizure-free was found in the edge connecting the right thalamic and the left supramarginal node (increased AD) and in edges connecting the left insula with frontal nodes (decreased FA). Patients who became seizure-free showed the largest effect size difference in the edge connecting the left isthmus cingulate and lateral occipital nodes (decreased FA).

One study related altered thalamocortical circuits to disorder severity and demonstrated a relationship between the extent of impaired connectivity and the number of generalized seizures in juvenile myoclonic epilepsy (O'Muircheartaigh et al., 2012). Caciagli et al., 2020 have proposed altered thalamic functional profiles as imaging biomarkers of active secondary generalization. Sinha et al. (2020, PREPRINT) have demonstrated greater and more widespread structural network alteration in patients with temporal lobe epilepsy and FBTCS when compared to patients without FBTCS. This is supported by our results as patients with FBTCS showed bilateral alterations in all metrics and more widespread bilateral decreased QA and more white matter alterations in edges involving the (left) thalamus and frontal, temporal and parietal nodes when compared to patients without FBTCS.

One methodological limitation in this multiple b-shell diffusion study pertains to the relatively low isotropic voxel spatial resolution. It was necessary to upsample our data to allow tractography as previously reported (Ahmadi et al., 2009, Yeatman et al., 2012). As there is no consensus or standard optimal parcellation scheme for connectomics, and there is no a priori hypothesis as to which parcellation is most appropriate, we chose the commonly used Desikan-Killiany atlas as in other epilepsy studies (Taylor et al., 2018, Taylor et al., 2015, Munsell et al., 2015). Nevertheless, we have also used a higher resolution parcellation scheme (Taylor et al., 2017, Besson et al., 2014b) in supplementary analysis. Compared to the analysis using the Desikan-Killiany atlas we have found similar results using the Destrieux atlas with respect to decreased anisotropy and increased diffusivity, although different connectomes have been shown to be significant. This was expected since the small sample size and higher atlas resolution will affect the entire connectome, which can ultimately lead to sparse connectomes and loss of statistical power. It is likely that parcellation schemes providing significantly more nodes may result in different connectomic alterations and may help to identify imaging network markers of pharmacoresistance in larger samples. There are also some limitations of our study that are an inherent reflection of studying patients with epilepsy at the time of diagnosis, which include: (a) a limited sample size as patients with a new diagnosis of epilepsy may not be seen at epilepsy specialist centers, (b) incomplete outcome data and (c) inclusion of patients with different foci (as focus localisation is infrequently ascertainable at the time of diagnosis). It is difficult and frequently impossible to determine seizure foci at diagnosis because of the few – single in many cases – epileptic events based on patient / witness testimony and unrevealing inter-ictal EEG (Kim et al., 2006). This is in contrast to longstanding refractory focal epilepsy where multiple imaging and clinical investigations have been conducted and from which a detailed picture of the likely seizure focus can be derived (Leek et al., 2020, Taylor et al., 2018). Nevertheless, our study revealed evidence of early disrupted structural networks in patients with NDfE who were deemed to be MRI-negative by expert neuroradiologists. This is an important finding demonstrating the usefulness of connectomics in studying the earliest stages of human epilepsy.

In conclusion, our findings indicate that structural brain connectivity is impaired in patients with epilepsy at the time of diagnosis. Furthermore, the extent of impairment may be related to seizure severity. This suggests that such impairments may be established prior to the onset of habitual seizures and do not necessarily result from the chronicity of the disorder or long-term AED use. Data presented here also indicates that a structural network marker of pharmacoresistance may be identifiable at the time of epilepsy diagnosis. It is possible that further network reconfiguration occurs in response to uncontrolled seizures; larger longitudinal imaging studies from the point of epilepsy diagnosis are needed to provide deeper insights.

## Sources of funding

This work was funded by an 10.13039/501100000295Epilepsy Research UK award to SSK and PNT (Grant number 1085). SSK also acknowledges support from the UK Medical Research Council (MR/S00355X/1 and MR/K023152/1) and PNT from the 10.13039/100010269Wellcome Trust (105617/Z/14/Z and 210109/Z/18/Z).

## Disclosure

None of the authors has any conflict of interest to disclose. We confirm that we have read the Journal’s position on issues involved in ethical publication and affirm that this report is consistent with those guidelines.

## CRediT authorship contribution statement

**Barbara A.K. Kreilkamp:** Conceptualization, Methodology, Software, Writing - original draft, Writing - review & editing, Validation. **Andrea McKavanagh:** Conceptualization, Methodology, Data curation, Software. **Batil Alonazi:** Data curation, Methodology. **Lorna Bryant:** Data curation, Methodology. **Kumar Das:** Conceptualization, Data curation, Methodology. **Udo C. Wieshmann:** Conceptualization, Data curation, Methodology. **Anthony G. Marson:** Conceptualization, Data curation, Methodology. **Peter N. Taylor:** Conceptualization, Methodology, Writing - review & editing, Software, Supervision. **Simon S. Keller:** Conceptualization, Methodology, Writing - review & editing, Validation, Supervision.

## Declaration of Competing Interest

The authors declare that they have no known competing financial interests or personal relationships that could have appeared to influence the work reported in this paper.
