# Supplementary material for: Altered structural connectome in non-lesional newly diagnosed focal epilepsy: Relation to pharmacoresistance
Source: Neuroimage Clin. 2021 Jan 19;29:102564. doi: 10.1016/j.nicl.2021.102564 (PMC7841400; doi:10.1016/j.nicl.2021.102564)
Supplement: Supplementary Data 1 [file mmc1.doc]

**Supplementary Materials**

|  | **PS vs Controls** | | |  | **SF vs Controls** | | |  | **PS vs SF** | | |
| --- | --- | --- | --- | --- | --- | --- | --- | --- | --- | --- | --- |
| **Metric** | node1 | node2 | Cohen's D |  | node1 | node2 | Cohen's D |  | node1 | node2 | Cohen's D |
| **QA** | **rh.thalamus**  rh.medialorbitofrontal  rh.parsorbitalis  rh.rostralmiddlefrontal  rh.rostralmiddlefrontal  rh.bankssts  rh.putamen  rh.superiorparietal  rh.putamen  rh.posteriorcingulate  rh.superiorparietal | rh.superiorparietal  lh.inferiortemporal  lh.medialorbitofrontal  rh.precuneus  rh.parsorbitalis  lh.pallidum  **rh.thalamus**  rh.rostralmiddlefrontal  rh.precuneus  lh.rostralmiddlefrontal  rh.precuneus | -1.00  -1.00  -1.05  -1.05  -1.06  -1.07  -1.10  -1.13  -1.13  -1.28  -1.40 |  | rh.precuneus  rh.lateralorbitofrontal  rh.caudate  rh.caudalanteriorcingulate  rh.frontalpole  rh.hippocampus  rh.hippocampus  rh.amygdala  lh.pallidum  rh.caudalmiddlefrontal  rh.postcentral  lh.transversetemporal  rh.accumbens  rh.putamen  rh.caudate  rh.medialorbitofrontal  lh.transversetemporal  **rh.thalamus**  **rh.thalamus**  rh.caudate  rh.bankssts | rh.parsorbitalis  lh.parsorbitalis  rh.superiorparietal  rh.bankssts  lh.amygdala  rh.superiorparietal  rh.putamen  rh.caudate  lh.inferiortemporal  lh.superiorparietal  rh.medialorbitofrontal  lh.temporalpole  rh.pericalcarine  rh.middletemporal  **rh.thalamus**  lh.pallidum  lh.rostralanteriorcingulate  rh.postcentral  rh.middletemporal  rh.middletemporal  **lh.thalamus** | -1.01  -1.02  -1.03  -1.04  -1.04  -1.08  -1.09  -1.09  -1.10  -1.11  -1.14  -1.14  -1.17  -1.17  -1.21  -1.26  -1.29  -1.31  -1.33  -1.33  -1.40 |  | rh.bankssts  rh.caudate  rh.caudalmiddlefrontal  rh.lateralorbitofrontal  rh.pallidum  rh.caudalmiddlefrontal  rh.medialorbitofrontal  rh.postcentral  lh.putamen  rh.lingual  **rh.thalamus**  rh.bankssts  rh.posteriorcingulate  rh.bankssts  rh.caudate  rh.posteriorcingulate  rh.putamen  rh.parstriangularis  rh.rostralmiddlefrontal | **lh.thalamus**  rh.middletemporal  lh.superiorparietal  lh.parsorbitalis  rh.transversetemporal  rh.bankssts  lh.pallidum  rh.medialorbitofrontal  **lh.thalamus**  lh.medialorbitofrontal  rh.parahippocampal  lh.postcentral  lh.rostralmiddlefrontal  lh.pallidum  rh.insula  rh.parsopercularis  rh.supramarginal  lh.insula  rh.lateraloccipital | 1.95  1.51  1.23  1.21  1.21  1.16  1.15  1.12  1.09  1.03  1.02  1.01  -1.05  -1.08  -1.11  -1.17  -1.21  -1.54  -2.26 |
| **FA** | rh.lateralorbitofrontal  rh.lateralorbitofrontal  rh.caudate  rh.precuneus  rh.transversetemporal  rh.putamen  rh.posteriorcingulate  **lh.thalamus**  rh.transversetemporal  **rh.thalamus**  rh.bankssts  rh.medialorbitofrontal  rh.superiorparietal | lh.parsorbitalis  rh.bankssts  rh.insula  rh.lateraloccipital  lh.isthmuscingulate  rh.precuneus  lh.rostralmiddlefrontal  lh.supramarginal  lh.inferiortemporal  rh.superiorparietal  lh.pallidum  lh.inferiortemporal  rh.precuneus | 1.09  1.02  -1.03  -1.05  -1.05  -1.07  -1.08  -1.12  -1.19  -1.22  -1.23  -1.41  -1.62 |  | rh.superiorfrontal  rh.caudate  **rh.thalamus**  lh.entorhinal  **lh.thalamus**  lh.posteriorcingulate  rh.caudalmiddlefrontal  rh.putamen  rh.medialorbitofrontal  rh.postcentral  lh.transversetemporal  **rh.thalamus**  rh.caudate  rh.caudalanteriorcingulate  rh.bankssts | lh.insula  rh.middletemporal  rh.postcentral  lh.bankssts  lh.supramarginal  lh.entorhinal  lh.superiorparietal  rh.middletemporal  lh.pallidum  rh.medialorbitofrontal  lh.temporalpole  rh.middletemporal  **rh.thalamus**  rh.bankssts  **lh.thalamus** | 1.01  -1.02  -1.03  -1.05  -1.06  -1.06  -1.07  -1.19  -1.21  -1.23  -1.32  -1.32  -1.33  -1.38  -1.42 |  | lh.lateraloccipital  rh.postcentral  rh.bankssts  rh.lateralorbitofrontal  rh.caudate  rh.caudalmiddlefrontal  rh.caudalmiddlefrontal  rh.caudalmiddlefrontal  rh.medialorbitofrontal  rh.entorhinal  rh.bankssts  rh.postcentral  lh.rostralanteriorcingulate  rh.bankssts  lh.putamen  rh.lateralorbitofrontal  rh.putamen  rh.putamen  lh.amygdala  lh.cuneus  rh.supramarginal  rh.supramarginal  rh.medialorbitofrontal  rh.putamen  rh.bankssts  rh.superiortemporal  rh.isthmuscingulate  rh.putamen  lh.superiorfrontal  rh.transversetemporal  rh.rostralmiddlefrontal  rh.posteriorcingulate  rh.posteriorcingulate  rh.caudate  rh.parstriangularis  rh.superiorfrontal | lh.isthmuscingulate  rh.medialorbitofrontal  **lh.thalamus**  lh.parsorbitalis  rh.middletemporal  lh.superiorparietal  **lh.thalamus**  rh.bankssts  lh.pallidum  lh.entorhinal  lh.rostralmiddlefrontal  rh.parahippocampal  lh.entorhinal  lh.postcentral  **lh.thalamus**  rh.bankssts  rh.caudate  rh.middletemporal  lh.paracentral  lh.caudalmiddlefrontal  lh.supramarginal  rh.rostralmiddlefrontal  lh.inferiortemporal  rh.precuneus  lh.pallidum  lh.supramarginal  lh.amygdala  rh.supramarginal  lh.bankssts  lh.inferiortemporal  rh.lateraloccipital  lh.rostralmiddlefrontal  rh.parsopercularis  rh.insula  lh.insula  lh.insula | 2.26  1.67  1.57  1.40  1.34  1.32  1.17  1.15  1.12  1.09  1.06  1.06  1.05  1.05  1.04  1.03  1.01  1.00  -1.01  -1.02  -1.04  -1.09  -1.10  -1.12  -1.17  -1.24  -1.26  -1.27  -1.32  -1.34  -1.41  -1.59  -1.61  -1.71  -1.90  -2.51 |
| **MD** | **lh.thalamus**  rh.posteriorcingulate  rh.parsopercularis  rh.putamen  rh.superiorparietal  **rh.thalamus**  rh.lingual  rh.transversetemporal  rh.putamen  lh.lingual  **rh.thalamus**  rh.insula  rh.superiorparietal  lh.rostralmiddlefrontal  **rh.thalamus**  rh.parahippocampal  rh.medialorbitofrontal | lh.supramarginal  lh.rostralmiddlefrontal  lh.pericalcarine  rh.supramarginal  rh.precuneus  lh.supramarginal  lh.rostralmiddlefrontal  lh.inferiortemporal  rh.precuneus  lh.entorhinal  rh.superiorparietal  lh.pallidum  rh.rostralmiddlefrontal  lh.posteriorcingulate  rh.middletemporal  rh.middletemporal  lh.rostralmiddlefrontal | 1.61  1.57  1.50  1.47  1.42  1.40  1.34  1.18  1.15  1.14  1.12  1.10  1.10  1.03  1.01  1.01  1.00 |  | rh.superiorfrontal  lh.pericalcarine  rh.putamen  lh.transversetemporal  rh.parsopercularis  lh.superiorfrontal  lh.postcentral  rh.caudalmiddlefrontal  rh.caudate  rh.caudalanteriorcingulate  lh.entorhinal  rh.postcentral  rh.postcentral  rh.fusiform  **rh.thalamus**  rh.caudalmiddlefrontal  lh.rostralmiddlefrontal  rh.insula  rh.inferiorparietal  lh.rostralmiddlefrontal  rh.caudate  rh.parstriangularis  rh.medialorbitofrontal  lh.lingual  rh.medialorbitofrontal  rh.fusiform | rh.medialorbitofrontal  lh.entorhinal  rh.middletemporal  lh.rostralanteriorcingulate  lh.pericalcarine  lh.middletemporal  lh.parahippocampal  lh.parahippocampal  rh.superiorparietal  rh.bankssts  lh.bankssts  rh.medialorbitofrontal  rh.middletemporal  rh.caudalmiddlefrontal  rh.middletemporal  lh.superiorparietal  lh.pericalcarine  rh.temporalpole  **lh.thalamus**  lh.posteriorcingulate  rh.rostralanteriorcingulate  lh.posteriorcingulate  lh.pallidum  lh.inferiortemporal  lh.pericalcarine  **lh.thalamus** | 1.54  1.46  1.34  1.31  1.26  1.22  1.19  1.19  1.15  1.15  1.15  1.15  1.14  1.14  1.13  1.12  1.10  1.09  1.08  1.07  1.06  1.04  1.04  1.03  1.02  1.02 |  | **rh.thalamus**  rh.posteriorcingulate  rh.insula  rh.precuneus  lh.superiortemporal  rh.lingual  rh.superiorparietal  rh.transversetemporal  rh.posteriorcingulate  rh.putamen  lh.caudate  rh.bankssts  rh.pallidum  rh.amygdala  rh.rostralmiddlefrontal  lh.superiortemporal  rh.caudate  rh.temporalpole  rh.inferiorparietal  lh.pericalcarine  rh.postcentral  rh.pericalcarine  rh.rostralanteriorcingulate  rh.inferiorparietal  rh.postcentral  rh.cuneus  rh.superiorfrontal  rh.caudalmiddlefrontal | lh.supramarginal  lh.rostralmiddlefrontal  lh.precentral  rh.lateraloccipital  lh.parstriangularis  lh.rostralmiddlefrontal  lh.supramarginal  lh.inferiortemporal  rh.parsopercularis  rh.supramarginal  lh.precentral  lh.supramarginal  rh.parsorbitalis  lh.superiorfrontal  rh.lateraloccipital  lh.rostralmiddlefrontal  rh.insula  lh.paracentral  rh.caudalanteriorcingulate  lh.entorhinal  rh.medialorbitofrontal  lh.inferiorparietal  rh.postcentral  rh.entorhinal  rh.parahippocampal  lh.superiorfrontal  rh.medialorbitofrontal  lh.parahippocampal | 2.13  1.74  1.67  1.32  1.31  1.24  1.23  1.20  1.13  1.13  1.13  1.13  1.09  1.07  1.04  1.02  1.01  -1.03  -1.05  -1.06  -1.09  -1.13  -1.13  -1.14  -1.14  -1.14  -1.17  -1.20 |
| **RD** | rh.posteriorcingulate  **lh.thalamus**  rh.superiorparietal  rh.parsopercularis  rh.putamen  rh.lingual  rh.transversetemporal  **rh.thalamus**  rh.putamen  **rh.thalamus**  rh.caudate  rh.superiorparietal  lh.lingual  rh.bankssts  lh.rostralmiddlefrontal  lh.rostralmiddlefrontal  rh.caudate  **rh.thalamus** | lh.rostralmiddlefrontal  lh.supramarginal  rh.precuneus  lh.pericalcarine  rh.supramarginal  lh.rostralmiddlefrontal  lh.inferiortemporal  lh.supramarginal  rh.precuneus  rh.superiorparietal  rh.insula  rh.rostralmiddlefrontal  lh.entorhinal  lh.pallidum  lh.posteriorcingulate  lh.pericalcarine  rh.rostralanteriorcingulate  rh.middletemporal | 1.58  1.57  1.51  1.48  1.46  1.37  1.30  1.25  1.19  1.16  1.12  1.09  1.08  1.08  1.03  1.03  1.01  1.00 |  | rh.putamen  rh.superiorfrontal  lh.pericalcarine  lh.transversetemporal  rh.postcentral  rh.parsopercularis  rh.caudalmiddlefrontal  lh.superiorfrontal  lh.entorhinal  rh.caudalanteriorcingulate  **rh.thalamus**  rh.caudate  lh.postcentral  rh.fusiform  lh.rostralmiddlefrontal  rh.caudalmiddlefrontal  rh.medialorbitofrontal  lh.lingual  rh.postcentral  rh.insula  rh.medialorbitofrontal  rh.putamen  rh.caudate  rh.inferiorparietal  lh.lateraloccipital  rh.fusiform  rh.accumbens  rh.putamen  rh.parstriangularis  rh.putamen | rh.middletemporal  rh.medialorbitofrontal  lh.entorhinal  lh.rostralanteriorcingulate  rh.medialorbitofrontal  lh.pericalcarine  lh.superiorparietal  lh.middletemporal  lh.bankssts  rh.bankssts  rh.middletemporal  rh.superiorparietal  lh.parahippocampal  rh.caudalmiddlefrontal  lh.pericalcarine  lh.parahippocampal  lh.pallidum  lh.inferiortemporal  rh.middletemporal  rh.temporalpole  lh.pericalcarine  **rh.thalamus**  rh.rostralanteriorcingulate  **lh.thalamus**  lh.isthmuscingulate  **lh.thalamus**  rh.supramarginal  rh.caudate  lh.posteriorcingulate  rh.postcentral | 1.54  1.47  1.46  1.36  1.30  1.26  1.23  1.22  1.22  1.22  1.21  1.21  1.18  1.16  1.15  1.15  1.08  1.07  1.06  1.05  1.05  1.04  1.04  1.04  1.04  1.03  1.02  1.01  1.01  1.01 |  | rh.posteriorcingulate  **rh.thalamus**  rh.insula  rh.transversetemporal  rh.precuneus  rh.posteriorcingulate  lh.superiortemporal  rh.lingual  rh.caudate  rh.putamen  rh.rostralmiddlefrontal  rh.pallidum  rh.amygdala  **rh.thalamus**  rh.bankssts  rh.bankssts  rh.inferiorparietal  lh.putamen  rh.temporalpole  rh.pericalcarine  rh.caudate  rh.inferiorparietal  lh.pericalcarine  rh.rostralanteriorcingulate  rh.caudalmiddlefrontal  rh.superiorfrontal  rh.postcentral  rh.postcentral | lh.rostralmiddlefrontal  lh.supramarginal  lh.precentral  lh.inferiortemporal  rh.lateraloccipital  rh.parsopercularis  lh.parstriangularis  lh.rostralmiddlefrontal  rh.insula  rh.supramarginal  rh.lateraloccipital  rh.parsorbitalis  lh.superiorfrontal  rh.superiorparietal  lh.pallidum  lh.supramarginal  rh.caudalanteriorcingulate  **lh.thalamus**  lh.paracentral  lh.inferiorparietal  rh.middletemporal  rh.entorhinal  lh.entorhinal  rh.postcentral  lh.parahippocampal  rh.medialorbitofrontal  rh.parahippocampal  rh.medialorbitofrontal | 1.78  1.78  1.44  1.38  1.37  1.34  1.24  1.22  1.20  1.19  1.16  1.06  1.03  1.02  1.01  1.01  -1.02  -1.05  -1.07  -1.08  -1.14  -1.15  -1.15  -1.17  -1.18  -1.20  -1.32  -1.45 |
| **AD** | **rh.thalamus**  rh.insula  rh.posteriorcingulate  rh.parsopercularis  rh.putamen  **lh.thalamus**  rh.lingual  lh.lingual  rh.superiorparietal  rh.medialorbitofrontal  rh.parstriangularis  rh.accumbens  lh.superiortemporal  rh.caudate  **rh.thalamus** | lh.supramarginal  lh.pallidum  lh.rostralmiddlefrontal  lh.pericalcarine  rh.supramarginal  lh.supramarginal  lh.rostralmiddlefrontal  lh.entorhinal  rh.precuneus  lh.rostralmiddlefrontal  rh.parsopercularis  rh.amygdala  lh.rostralmiddlefrontal  rh.inferiortemporal  rh.middletemporal | 1.59  1.53  1.50  1.47  1.45  1.39  1.20  1.14  1.11  1.10  1.07  1.02  1.01  1.01  1.01 |  | rh.superiorfrontal  lh.pericalcarine  rh.putamen  rh.postcentral  rh.parsopercularis  lh.postcentral  rh.caudalmiddlefrontal  lh.rostralmiddlefrontal  rh.fusiform  rh.parstriangularis  rh.insula  lh.transversetemporal  rh.accumbens  rh.caudate  lh.superiorfrontal  rh.inferiorparietal | rh.medialorbitofrontal  lh.entorhinal  rh.precuneus  rh.middletemporal  lh.pericalcarine  lh.parahippocampal  lh.parahippocampal  lh.posteriorcingulate  rh.caudalmiddlefrontal  rh.parsopercularis  rh.temporalpole  lh.rostralanteriorcingulate  rh.amygdala  rh.rostralanteriorcingulate  lh.middletemporal  **lh.thalamus** | 1.45  1.28  1.25  1.22  1.18  1.18  1.16  1.13  1.07  1.07  1.06  1.06  1.06  1.05  1.03  1.01 |  | **rh.thalamus**  rh.posteriorcingulate  lh.superiortemporal  rh.insula  rh.frontalpole  rh.bankssts  rh.superiorparietal  rh.lingual  lh.superiortemporal  lh.caudate  rh.superiorparietal  rh.amygdala  rh.pallidum  rh.superiorfrontal  rh.pericalcarine  rh.inferiorparietal  rh.inferiorparietal  rh.pallidum  rh.caudalmiddlefrontal  rh.parsopercularis  rh.cuneus  rh.accumbens | lh.supramarginal  lh.rostralmiddlefrontal  lh.parstriangularis  lh.precentral  lh.supramarginal  lh.supramarginal  lh.supramarginal  lh.rostralmiddlefrontal  lh.rostralmiddlefrontal  lh.precentral  rh.precentral  lh.superiorfrontal  rh.parsorbitalis  rh.medialorbitofrontal  lh.inferiorparietal  rh.entorhinal  rh.caudalanteriorcingulate  rh.insula  lh.parahippocampal  lh.insula  lh.superiorfrontal  rh.transversetemporal | 2.52  1.64  1.41  1.32  1.27  1.26  1.24  1.23  1.19  1.16  1.14  1.03  1.01  -1.00  -1.05  -1.10  -1.11  -1.12  -1.14  -1.22  -1.33  -1.35 |

**Table 1, Suppl.** Cohen's D values for edges between two nodes (node1 and node2) with weights QA, FA, MD, RD, AD for subgroup comparisons.

*Note.* Cohen's D values include the correction for small sample sizes. The thalamus nodes (left and right) have been highlighted in boldface, other nodes that are part of the altered thalamic network in patients with persistent seizures (PS) compared to patients who became seizure-free (SF) have been underlined. QA = quantitative anisoptropy; FA = fractional anisotropy; MD = mean diffusivity; RD = radial diffusivity; AD = axial diffusivity; rh = right hemispheric; lh = left hemispheric.

| **FBTCS vs NoFBTCS** | | | | | | | | | | |
| --- | --- | --- | --- | --- | --- | --- | --- | --- | --- | --- |
| **QA** | | |  | **FA** | | |  | **MD** | | |
| node1 | node2 | Cohen's D |  | node1 | node2 | Cohen's D |  | node1 | node2 | Cohen's D |
| rh.parsopercularis  rh.parstriangularis  rh.precuneus  rh.inferiorparietal  rh.precuneus  rh.inferiorparietal  rh.precentral  rh.isthmuscingulate  rh.temporalpole  rh.insula  rh.inferiorparietal  rh.caudalmiddlefrontal  rh.fusiform  rh.supramarginal  rh.caudalmiddlefrontal  rh.caudalmiddlefrontal  rh.posteriorcingulate  lh.parstriangularis  rh.parsorbitalis  rh.bankssts  rh.parahippocampal  lh.precentral  rh.precentral  rh.superiorparietal  lh.fusiform | rh.lateralorbitofrontal  lh.precentral  rh.parsorbitalis  lh.temporalpole  rh.postcentral  rh.entorhinal  lh.caudate  lh.supramarginal  rh.frontalpole  rh.parsorbitalis  lh.transversetemporal  **lh.thalamus**  **lh.thalamus**  rh.precentral  rh.bankssts  lh.postcentral  lh.lingual  lh.entorhinal  lh.inferiortemporal  lh.superiorparietal  rh.lingual  lh.parsorbitalis  lh.paracentral  lh.inferiortemporal  lh.entorhinal | 1.12  -1.00  -1.00  -1.00  -1.02  -1.03  -1.03  -1.05  -1.07  -1.08  -1.08 -1.09  -1.09  -1.09  -1.10  -1.13 -1.15  -1.16  -1.20  -1.23  -1.23  -1.29  -1.30  -1.37 -1.42 |  | rh.parstriangularis  rh.transversetemporal  rh.superiorfrontal  rh.superiorfrontal  lh.accumbens  rh.parsopercularis  rh.pericalcarine  rh.inferiorparietal  lh.precentral  rh.amygdala  lh.precuneus  rh.paracentral  rh.temporalpole  rh.caudalmiddlefrontal  rh.caudalmiddlefrontal  rh.isthmuscingulate  rh.bankssts  rh.fusiform  rh.insula  lh.parstriangularis  rh.precuneus  rh.superiorparietal  rh.parahippocampal | lh.insula  rh.precuneus  lh.insula  lh.pallidum  lh.paracentral  rh.lateralorbitofrontal  rh.cuneus  rh.caudalmiddlefrontal  lh.entorhinal  rh.pericalcarine  lh.postcentral  rh.bankssts  lh.caudate  **lh.thalamus**  lh.postcentral  lh.caudate  lh.superiorparietal  **lh.thalamus**  rh.paracentral  lh.entorhinal  rh.parsorbitalis  lh.inferiortemporal  rh.lingual | 1.41  1.30  1.26  1.21  1.17  1.14  1.09  1.07  1.06  1.05  1.05  1.04  -1.01  -1.04  -1.04  -1.09  -1.11  -1.13  -1.14  -1.14  -1.25  -1.35  -1.51 |  | rh.precentral  **lh.thalamus**  lh.temporalpole  lh.lateraloccipital  rh.entorhinal  rh.rostralanteriorcingulate  rh.pericalcarine  lh.superiortemporal  **rh.thalamus**  rh.lingual  rh.entorhinal  rh.amygdala  lh.rostralanteriorcingulate  lh.precentral  lh.insula  rh.transversetemporal  rh.rostralanteriorcingulate  rh.entorhinal  lh.lingual | lh.paracentral  lh.frontalpole  lh.rostralanteriorcingulate  lh.inferiortemporal  lh.temporalpole  rh.postcentral  lh.inferiorparietal  lh.parstriangularis  lh.supramarginal  lh.pericalcarine  lh.bankssts  rh.lingual  lh.entorhinal  lh.entorhinal  lh.pericalcarine  rh.precuneus  rh.parsorbitalis  lh.rostralmiddlefrontal  lh.entorhinal | 1.12  1.12  1.11  1.11  1.10  1.04  1.02  -1.00  -1.01  -1.03  -1.06  -1.10  -1.12  -1.16  -1.18  -1.32  -1.37  -1.39  -1.48 |
|  |  |  |  |  |  |  |  |  |  |  |
| **RD** | | |  | **AD** | | |  |  |  |  |
| node1 | node2 | Cohen's D |  | node1 | node2 | Cohen's D |  |  |  |  |
| rh.precentral  lh.lateraloccipital  **lh.thalamus**  rh.rostralanteriorcingulate  lh.temporalpole  rh.bankssts  rh.entorhinal  rh.parahippocampal  rh.entorhinal  lh.superiortemporal  lh.insula  rh.paracentral  lh.rostralanteriorcingulate  rh.amygdala  lh.precentral  rh.rostralanteriorcingulate  rh.entorhinal  rh.transversetemporal  lh.lingual | lh.paracentral  lh.inferiortemporal  lh.frontalpole  rh.postcentral  lh.rostralanteriorcingulate  lh.superiorparietal  lh.temporalpole  rh.lingual  lh.bankssts  lh.parstriangularis  lh.pericalcarine  rh.bankssts  lh.entorhinal  rh.lingual  lh.entorhinal  rh.parsorbitalis  lh.rostralmiddlefrontal  rh.precuneus  lh.entorhinal | 1.15  1.11  1.09  1.07  1.07  1.05  1.02  1.00  -1.00  -1.02  -1.05  -1.05  -1.09  -1.11  -1.19  -1.29  -1.37  -1.44  -1.52 |  | rh.entorhinal  lh.temporalpole  lh.rostralmiddlefrontal  lh.lateraloccipital  **lh.thalamus**  rh.pericalcarine  rh.postcentral  lh.superiortemporal  rh.amygdala  **rh.thalamus**  lh.precentral  rh.inferiorparietal  lh.rostralanteriorcingulate  rh.entorhinal  rh.paracentral  rh.entorhinal  rh.entorhinal  rh.superiorparietal  rh.lingual  rh.rostralanteriorcingulate  lh.insula | lh.temporalpole  lh.rostralanteriorcingulate  lh.posteriorcingulate  lh.inferiortemporal  lh.frontalpole  lh.inferiorparietal  rh.parahippocampal  lh.rostralmiddlefrontal  rh.lingual  rh.insula  lh.entorhinal  lh.superiorfrontal  lh.entorhinal  lh.entorhinal  lh.pericalcarine  lh.rostralmiddlefrontal  lh.bankssts  rh.precentral  lh.pericalcarine  rh.parsorbitalis  lh.pericalcarine | 1.18  1.14  1.09  1.07  1.05  1.04  1.02  -1.01  -1.03  -1.05  -1.09  -1.09  -1.09  -1.10  -1.11  -1.12  -1.13  -1.19  -1.27  -1.31  -1.35 |  |  |  |  |

**Table 2, Suppl.** Cohen's D values for edges between two nodes (node1 and node2) with weights QA, FA, MD, RD, AD for FBTCS and NoFBTCS subgroup comparisons.

*Note.* Cohen's D values include the correction for small sample sizes. The thalamus nodes (left and right) have been highlighted in boldface, other nodes that are part of the altered thalamic network in patients with FBTCS compared to patients without FBTCS have been underlined. QA = quantitative anisoptropy; FA = fractional anisotropy; MD = mean diffusivity; RD = radial diffusivity; AD = axial diffusivity; rh = right hemispheric; lh = left hemispheric; FBTCS = focal-to-bilateral tonic-clonic seizures; NoFBTCS = no focal-to-bilateral tonic-clonic seizures.

**Methods**

For generation of T1w gray matter segments, cortical and subcortical parcellations were computed using Freesurfer Version 6 with the Destrieux atlas (Destrieux et al. 2010). The gray and white matter boundary segmentations were manually corrected using the recommended FreeSurfer PialEdits/ControlPoints procedures before 148 cortical and 14 subcortical regions-of-interest were extracted and used as network nodes for connectomics. Disconnected nodes in individual participants were dilated into adjacent white matter (left accumbens, ctx_lh_S_orbital_lateral, ctx_rh_S_temporal_transverse and ctx_lh_S_interm_prim-Jensen) so they became part of the connectome. The diffusion processing and steps in dsi-studio were done as described in the main body of the paper. Matrix entries represented edges from tractography and were set as present if more than one streamline terminated in two nodes derived from segmentations. The average edge diffusion metrics were directly calculated from all streamlines connecting two nodes. Connectivity matrix thresholding was performed so that edges were present in at least 75% of all controls/patients (most common in sample) and edges were common to every group (Besson et al. 2014a).

**Results**

Patients showed decreased anisotropy and increased diffusivity (QA range =2.5/2.9-2.7; MD range=3.2/3.8-4.0; RD range=3.2-3.5/3.9-4.0) across multiple NBS thresholds relative to controls (Figure 1, Suppl), but no alterations in FA or AD. The edges with the largest |T|-scores for every metric are highlighted in red in Figure 1, Suppl and involved the right inferior supramarginal, left anterior insular and left inferior temporal nodes.


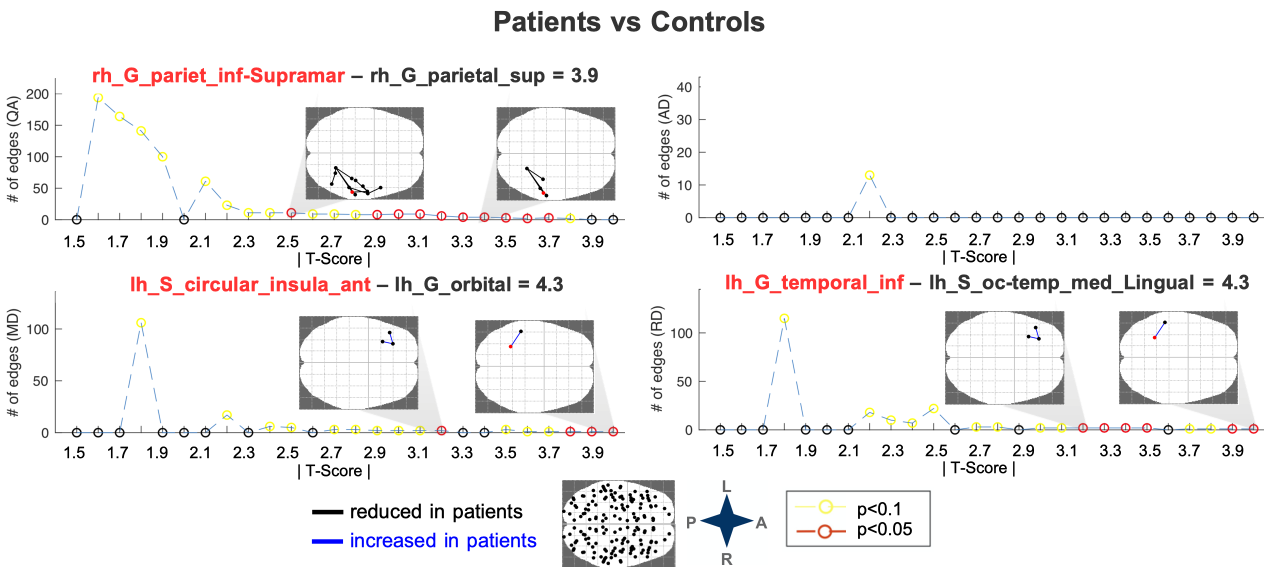


**Figure 1 (Suppl). Significant NBS networks in patients vs controls across different T-score thresholds.**

Networks were visualized with NBSview. Inset shows complete node set (N=162, Destrieux et al. 2010) used for analysis. NBS identified several edges for different T-scores and significant networks are displayed for selected exemplary T-scores for visualization purposes. Edges with the highest T-scores are denoted by highlighting the affected node in red along with the connected node and T-score in the subplot titles. L=left; R=right; A=anterior; P=posterior; rh=right hemisphere; lh=left hemisphere; QA=quantitative anisotropy; MD/AD/RD=mean/axial/radial diffusivity.
